# Supplementary material for: Splice-Junction-Based Mapping of Alternative Isoforms in the Human Proteome
Source: Cell Rep. Author manuscript; Available in PMC 2020 Jan 15. (PMC6961840; doi:10.1016/j.celrep.2019.11.026)
Supplement: 2 [file NIHMS1546469-supplement-2.zip › DF1/PXD006675/RightCoronaryArtery/RightCoronaryArtery_7_SRRM1_KVELSESEDK.pdf]

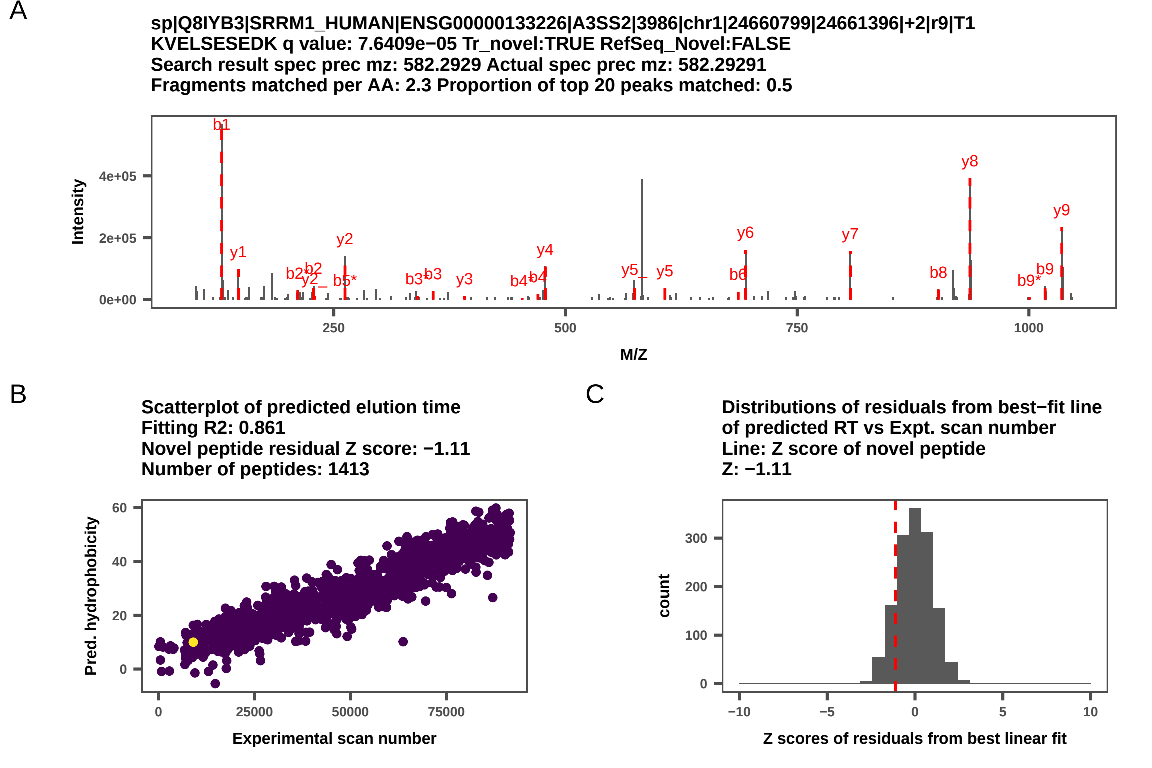
A
sp|Q8IYB3|SRRM1_HUMAN|ENSG00000133226|A3SS2|3986|chr1|24660799|24661396|+2|r9|T1
KVELSESEDK q value: 7.6409e−05 Tr_novel:TRUE RefSeq_Novel:FALSE
Search result spec prec mz: 582.2929 Actual spec prec mz: 582.29291
Fragments matched per AA: 2.3 Proportion of top 20 peaks matched: 0.5
Intensity
M/Z
B
Scatterplot of predicted elution time
Fitting R2: 0.861
Novel peptide residual Z score: −1.11
Number of peptides: 1413
Pred. hydrophobicity
Experimental scan number
C
Distributions of residuals from best−fit line
of predicted RT vs Expt. scan number
Line: Z score of novel peptide
Z: −1.11
count
Z scores of residuals from best linear fit
